# Do hospitals that participate in COVID-19 research differ from non-trial hospitals? A cross-sectional study of US hospitals

**DOI:** 10.1186/s13063-023-07450-6

**Published:** 2023-08-07

**Authors:** Daniel Kang, Cher X. Huang, Alexander D. Yuen, Keith C. Norris, Tara Vijayan

**Affiliations:** 1 University of Illinois, Urbana Champaign, Champaign, USA; 2 Department of Medicine, Massachusetts General Hospital, Boston, USA; 3 Department of Medicine, David Geffen School of Medicine, University of California, Los Angeles, USA; 4 Division of General Internal Medicine and Health Services Research, David Geffen School of Medicine, University of California, Los Angeles, USA; 5 Division of Infectious Diseases, David Geffen School of Medicine, University of California, Los Angeles, USA

## Abstract

**Objectives:**

To compare hospitals that did and did not participate in clinical trials evaluating potential inpatient COVID-19 therapeutics.

**Methods:**

We conducted a cross-sectional study of hospitals participating in trials that were registered on clinicaltrials.gov between April and August 2020. Using the 2019 RAND Hospital Dataset and 2019 American Community Survey, we used logistic regression modeling to compare hospital-level traits including demographic features between trial and non-trial hospitals.

**Results:**

We included 488 hospitals that were participating in 298 interventional trials and 4232 non-participating hospitals. After controlling for demographic and other hospital traits, we found that teaching status (OR 2.11, 95% CI 1.52–2.95), higher patient acuity (OR 7.48, 4.39, 13.1), and location in the Northeast (OR 1.83, 95% CI 1.18, 2.85) and in wealthier counties (OR: 1.32, 95% CI 1.16–1.51) were associated with increased odds of trial participation, while being in counties with more White residents was associated with reduced odds (OR 0.98, 95% CI 0.98–0.99).

**Conclusions:**

Hospitals participating and not participating in COVID-19 inpatient treatment clinical trials differed in many ways, resulting in important implications for the generalizability of trial data.

**Supplementary Information:**

The online version contains supplementary material available at 10.1186/s13063-023-07450-6.

## Background

The burden of critical illness from coronovirus disease 2019 (COVID-19) took a tremendous adverse toll on hospitals and health systems across the world as most struggled to care for infected patients who were disproportionately from marginalized racial and ethnic minority groups, while at the same time many patients with non-COVID-19 conditions suffered from delayed care. To address these negative outcomes, COVID-19 has heralded a massive effort to study novel pharmaceuticals and technological interventions to reduce mortality and to alter disease course among hospitalized patients [1]. Many of these studies are randomized controlled trials (RCTs), the gold standard for evaluating safety and efficacy [2]. However, these trials have limitations, including the diversity of included patients [3]. Patient diversity in clinical trials has important implications for COVID-19 due to the well-documented excess burden of morbidity and mortality experienced by minority groups [4]. Indeed, recent articles have highlighted geographic disparities in access to COVID-19 clinical trials [5, 6].

Less is known about hospital-level disparities in access to clinical trials in pandemics. Thus, in this study, we describe the hospitals participating in COVID-19 inpatient clinical trials and assess whether there are differences between hospitals that did and did not participate in these trials.

## Methods

### Data sources

We conducted a cross-sectional study of hospitals participating in interventional trials evaluating inpatient COVID-19 treatment options registered on ClinicalTrials.gov between April 1 and August 24, 2020. We excluded trials evaluating outpatient therapeutics and vaccine trials. We matched this cohort to the 2019 RAND Hospital Dataset [7], a dataset collating characteristics submitted annually to the Centers for Medicare and Medicaid Services (CMS), including hospital size, patient acuity, and safety net status. We excluded Veteran Affairs hospitals and childrens’ hospitals as they were not available in the RAND dataset. We linked this cohort of hospitals to the 2015–2019 American Community Survey (ACS) [8], using the Federal Information Processing System (FIPS) codes to associate hospitals with the corresponding geographic counties’ population-level demographics [9] (Supplemental Fig. 1).

### Hospital characteristics

We included the following hospital characteristics: hospital size (the number of hospital beds), teaching status (affiliated with at least one residency training program), patient illness severity (average Medicare case-mix index), and percentage of low-income patients who received care at that hospital (Medicare disproportionate payment percentage) [10].

### Demographic characteristics

We obtained the following county-level variables from the ACS: US census region (Northeast, Midwest, South, West), median income (expressed in $10,000/year units), race (percentage White vs non-White), ethnicity (percentage of county residents who identify as Latinx), and urban–rural designation. The demographic information was limited to White vs non-White due to our sample size of hospitals.

### Statistical analysis

We used descriptive statistics to summarize the characteristics of hospitals that did (trial hospitals) and did not (non-trial hospitals) participate in clinical trials.

Using a multivariable logistic regression model, we assessed whether trial hospitals differed from non-trial hospitals according to the hospital and demographic variables while controlling for each other. For the descriptive statistics, we used the Wald test for each variable in the regression model. We corrected for the multiple hypotheses using the Bonferroni correction. Statistical analyses were conducted using Python 3.8.8 and R 3.5.1.

This study was exempt from review by the Institutional Review Board (IRB) as we exclusively reviewed publicly available information and did not review any individual patient records. This study was prepared in accordance with the STROBE reporting guidelines for cross-sectional studies [11].

## Results

Between April 1 and August 24, 2020, there were 541 hospitals participating in 298 randomized, clinical trials evaluating treatment options for patients hospitalized for COVID-19. After excluding hospitals not available in the RAND Hospital Dataset, there were 488 trial hospitals and 4232 non-trial hospitals. We believe these sites were missing because a small number of hospitals in the USA are not registered with the CMS and several medical centers listed in the clinicial trials are eligible to perform trials but are actually large clinics and not hospitals.

We found that 79.5% of trial hospitals were teaching hospitals, compared to 20.1% of non-trial hospitals. Trial hospitals were also larger (427 beds vs 110.8 beds, *p* < 0.001). The average case-mix index and disproportionate share percentage for trial hospitals were also higher compared to non-trial hospitals (Table 1). There were also demographic differences: trial hospitals were in counties that had higher annual median incomes ($3.19 vs $2.73), had a lower percentage of white residents, and had a higher percentage of Latinx residents.

**Table 1:** Characteristics of trial and non-trial hospitals

	Characteristics of hospitals			Adjusted odds of trial participation	
	Trial hospitals	Non-trial hospitals	*p*-value	OR	[95% CI]
*Hospital-level characteristics*					
% Teaching Status	79.5	20.1	< 0.001	2.112^***^	[1.520, 2.949]
Average number of hospital beds (SD)	427.0 (295.0)	110.8 (133.2)	< 0.001	1.003^***^	[1.003, 1.004]
Disproportionate Share Percentage	20.9	15.0	< 0.001	2.264	[0.755,6.687]
Average Medicare case-mix index (SD)	1.93 (0.32)	1.57 (0.37)	< 0.001	7.484^***^	[4.386, 13.057]
*Demographics characteristics*					
Median annual income (in $10,000)	$3.19	$2.73		1.320^***^	[1.158, 1,505]
Race: % White	64.1	79.3	< 0.001	0.983^***^	[0.977, 0.990]
Ethnicity: % Latinx	18.7	13.0	< 0.001	1.294	[0.574, 2.859]
Urban/rural:					
% Rural (reference)	5.3	45.4		1.000	
% Urban	94.7	54.6		1.815^*^	[1.079, 3.182]
US census region:					
% Northeast	22.7	11.0	< 0.001	1.825^***^	[1.175, 2.845]
% Midwest (ref)	16.8	30.9		1.000	
% South	29.9	36.2		0.981	[0.655, 1.475]
% West	27.9	19.0		1.600^*^	[1.023, 2.513]

^*^p < 0.05

^**^p < 0.001

We found that, after controlling for other demographic and hospital characteristics, teaching status (OR: 2.11, 95% CI: 1.52–2.95), having a greater number of hospital beds (OR 1.003, 95% CI: 1.003–1.004), and higher Medicare case-mix index (OR 7.48, 95% CI: 4.39–13.1) were independently associated with higher odds of trial participation at the hospital level. Similarly, being in counties with higher annual median incomes (OR 1.32, 95% CI: 1.16–1.51) and lower percentage of White residents (OR 0.98, 95% CI 0.98–0.99) as well as being in the Northeast census region (OR 1.83, 95% CI 1.18–2.85) were each independently associated with statistically significant higher odds of participating in a COVID-19 trial.

## Discussion

We found that early in the COVID-19 pandemic, there were multiple differences between trial and non-trial hospitals. We found differences in geographic access to trials, with hospitals in the Northeast having the highest odds of trial participation [5, 6]. Additionally, we found that larger size, teaching status, and locations in wealthier counties with fewer White residents were each independently associated with higher odds of trial participation during the early stage of the COVID-19 pandemic. We also found that higher patient acuity was also associated with increased odds of trial participation, which we believe is important for inpatient clinical trials focusing on severe disease.

Although we controlled for teaching status, we did not have a marker for major tertiary and quaternary academic centers. As such, some of the differences may be driven by access to academic medical centers. It is possible that academic medical centers may have been the best equipped to mobilize the resources necessary for trial participation [6]. This has important implications for the generalizability of trial results; efficacy and safety outcomes generated at these highly specialized centers may not necessarily be replicated in community centers with fewer resources. Other factors, such as the number of ICU beds and whether or not the hospital has an accident and emergency department, may also affect the presence of clinical trials, which we did not control for.

Our study has limitations. First, we used county-level demographic characteristics as a proxy for the demographics for each hospital’s patient population. While hospitals’ patient populations may not always reflect the surrounding county demographics, we felt that this was a suitable proxy in the setting of the COVID-19 pandemic, where patients may not be traveling as frequently to receive care from further hospitals. However, this may affect the validity of our analysis. Additionally, we limited our study sample to inpatient trials; our results may not be generalizable to trials evaluating outpatient therapeutics. Finally, COVID-19 policies ranging from non-pharmaceutical interventions to hospital policies varied greatly from region to region. For example, restaurant closures and mandatory masking were imposed at different times across regions. Our analysis does not take into account the spatial and temporal differences in these policies.

Our analysis did not find differences in racial compositions, which we believe is due to limitations of our data. Hospitals that participated in clinical trials are clustered in urban regions. These regions are more racially diverse, but hospitals do not necessarily serve the entire population in the geographic regions we considered. Furthermore, income inequality has risen substantially in these more densely populated urban regions such as San Francisco, Los Angeles, and New York City. While the disproportionate share was higher among hospitals that participated in trials, this does not necessarily mean that clinical trials included more individuals from lower socioeconomic backgrounds. In one of the earliest trials, ACTT-1, which studied the benefit of remdesivir among hospitalized patients, 43% of participants idenfied as Black or Hispanic, but data on income or insurance status were not reported [12]. We encourage clinical trials to release demographic information in standardized formats to allow further analysis to be done.

## Public health implications

In conclusion, we found that not only are there significant geographic differences in access to trials, there are also significant hospital-level differences, including teaching status, size, and location within wealthier counties. Trials remain heavily concentrated in urban areas, widening the rural–urban gap in access to care. These differences have important implications for the generalizability of trial data. Further efforts should promote the expansion of clinical trial sites to a more diverse set of hospitals, including those in low-income, rural communities such as those in the Southeast and Midwest, to increase generalizability of trial results.

### Supplementary Information

**Additional file 1: Supplemental Figure 1.** Distribution of Trials Across the US (map figure).

## Data Availability

The datasets generated in this current study are available from the RAND dataset, ClinicalTrials.gov, and the American Community Survey 2019 dataset. The linkages are available from the corresponding author on request.
