# Supplementary figures and images for: Do hospitals that participate in COVID-19 research differ from non-trial hospitals? A cross-sectional study of US hospitals
Source: Trials. 2023 Aug 7;24:504. doi: 10.1186/s13063-023-07450-6 (PMC10408090; doi:10.1186/s13063-023-07450-6)

**Supplemental Figure 1. Distribution of Trials Across the US (map figure)**


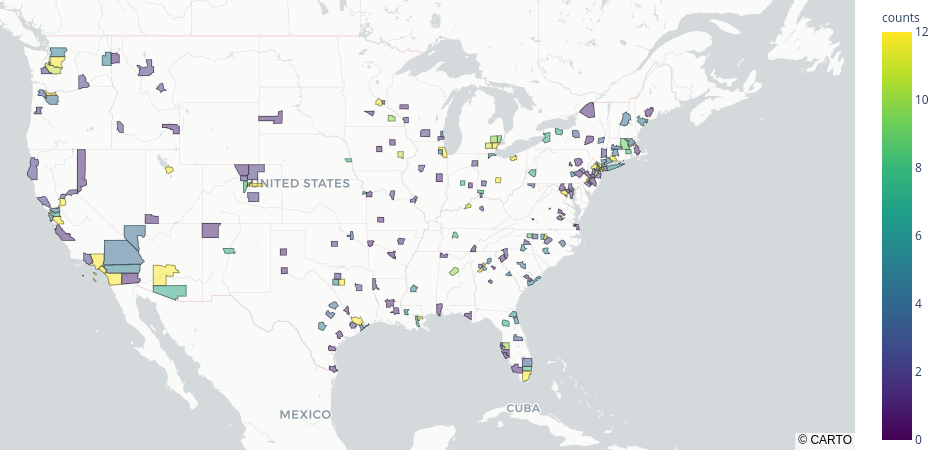

Supplement: Supplementary file 1 — Additional file 1: Supplemental Figure 1. Distribution of Trials Across the US (map figure). [file 13063_2023_7450_MOESM1_ESM.docx]
